# LEX-BADAT: Language EXperience in Bilinguals With and Without Aphasia DATaset

**DOI:** 10.3389/fpsyg.2022.875928

**Published:** 2022-06-13

**Authors:** Manuel Jose Marte, Erin Carpenter, Isaac B. Falconer, Michael Scimeca, Fatemeh Abdollahi, Claudia Peñaloza, Swathi Kiran

**Affiliations:** ^1^Aphasia Research Laboratory, Department of Speech, Language, and Hearing Sciences, Boston University, Boston, MA, United States; ^2^Department of Cognition, Development and Educational Psychology, Faculty of Psychology, University of Barcelona, Barcelona, Spain; ^3^Institute of Neurosciences, University of Barcelona, Barcelona, Spain; ^4^Cognition and Brain Plasticity Unit, Institut d'Investigació Biomèdica de Bellvitge (IDIBELL), L'Hospitalet de Llobregat, Barcelona, Spain

**Keywords:** bilingual aphasia, bilingualism, language experience, language use questionnaire, language history

## Introduction

Bilingualism is a gradient of experiences that show significant variation across individuals who speak more than one language (DeLuca et al., 2019). This inter-individual variation is evident along several axes between first- (L1) and second-acquired (L2) languages, including proficiency and daily usage, especially when considering unbalanced bilinguals. As the incidence of acquired brain injury (ABI), e.g., stroke, increases (Katan and Luft, 2018) leading to language impairment in aging bilingual populations, it can be expected that bilingual people with aphasia (BPWA) will comprise a greater share of caseloads in forthcoming years (Centeno et al., 2020).

Examining the influence of bilingual language experience on the performance of BPWA on linguistic and cognitive standardized assessments and experimental tasks remains an important pursuit to address basic research questions about language in the bilingual brain post-ABI, and to personalize care and predict individualized outcomes. However, understanding and quantifying language experience in bilingual individuals remains a highly complex undertaking (see Silva-Corvalán and Treffers-Daller, 2015; Köpke and Genevska-Hanke, 2018 for a review), a factor that is further complicated in individuals with ABI for whom language experiences may change following aphasia onset (Peñaloza et al., 2019). Several resources and assessments are often employed to elaborate dimensions of language experience (e.g., use, confidence, and proficiency) and address important questions in bilingual research (Francis, 2021). However, there is no clear consensus in the field on how to survey and quantify language experience in healthy bilinguals (HB) and BPWA, suggesting a need for consistency when collecting and reporting these measures across different studies (e.g., Kašćelan et al., 2022).

Finally, large-scale recruitment of BPWA is a challenging undertaking given the resources required to process participants through studies. As Kašćelan et al. (2022) suggest, bilingualism is an intricate construct and therefore variability among different approaches has arisen in operationalizing bilingualism for research purposes. Accordingly, transparency in methods and conceptualization of measures is relatively low, resulting in controversies such as the equivocal presence of bilingual advantage in executive function (Marian and Hayakawa, 2021). While various datasets exist relating to aphasia (e.g., Mirman et al., 2010; MacWhinney et al., 2011) and multilingualism (e.g., de Bruin et al., 2017) separately, few large datasets exist representative of both populations simultaneously. Furthermore, much of the presently available bilingual data lacks more detailed information. For example, Surrain and Luk (2019) examined 186 studies published between 2005 and 2015 to examine the features of bilingual experience reported. While 79% of studies reported general information about language use at home, a minority (39%) reported this using proportions. de Bruin (2019), reviewing different language history questionnaires and measurement tools, also found that for metrics like age of acquisition (AoA), proficiency, and language use, there was a significant lack of definitional congruency, e.g., “late” vs. “early” bilingualism.

To that end, the aim of this Data Report is to introduce LEX-BADAT: Language EXperience in Bilinguals with and without Aphasia DATaset. We used the language use questionnaire (LUQ; Kastenbaum et al., 2019)—which uses continuous scales and has shown predictive value regarding lexical access in both HB (Kastenbaum et al., 2019) and BPWA (Peñaloza et al., 2019)—and explicitly defined its metrics. We provide summaries of the two datasets included, one of 85 BPWA and one of 31 HB. Additionally, given that the data are multidimensional, we present the results of principal component analyses (PCA) on raw LUQ data for ease of use in statistical analyses (e.g., generating component scores for participants not included in the analyses can be accomplished using the provided loading matrices), and to detect latent variables summarizing bilingual language experience in these two groups. As a result, this is both (i) the largest dataset of language experience in Spanish-English BPWA, and (ii) the largest dataset that includes both HB and BPWA using a shared instrument with directly comparable scales. We believe these data meaningfully inform researchers about the structure of language experience in linguistically heterogenous populations primarily in the United States, and perhaps more importantly, on the underexplored language experience of bilinguals whose access to language is impaired.

## Data Description

### Participants

Data from 85 BPWA (age = 53.28 ± 15.74; L2 AoA = 13.46 ± 11.91) and 31 HB (age = 43.10 ± 15.59; L2 AoA = 13.77 ± 12.00) were collected across several studies (Kastenbaum et al., 2019; Peñaloza et al., 2019, 2020, 2021). As previously stated, participants were from linguistically heterogeneous populations primarily within the United States and also abroad. Distribution of BWPA included: 43 Northeast, 26 South and Southeast, 15 West Coast, and 1 Canada. Distribution of HB included: 24 Northeast, 3 West Coast, 1 Southeast, 1 Caribbean, and 1 Southern Europe. Spanish was reported as the L1 for 72 BPWA and for 27 HB. All BPWA were at least 1 month post-onset (MPO = 48.66 ± 73.16) and presented with aphasia secondary to stroke (*n* = 80), traumatic brain injury (*n* = 4), or brain tumor (*n* = 1). Lesion and clinical information for BPWA is noted in Supplementary Table 1. All participants provided their written consent for standardized language testing in accordance with procedures approved by the Ethical Committee of the University of Texas at Austin and Boston University.

### Language Use Questionnaire

The LUQ was originally designed for HB (Kastenbaum et al., 2019) and was later adapted for use in BPWA (Peñaloza et al., 2019, 2020, 2021) *via* addition of post-stroke queries. The LUQ obtained information about language experience metrics in L1 and L2. In the case of BPWA, the LUQ distinguished between pre- and post-ABI for applicable L1 and L2 language experience metrics to reflect changes in their language patterns between these two timepoints. LUQ measures used in this dataset included: *Age of Acquisition (AoA)* reflected the age at which participants reported having begun to learn their L2. We noted L2 *AoA* as 0 for simultaneous bilinguals. *Language Ability Rating (LAR)* captured participants' ratings of their L1 and L2 ability on a 5-point scale (1 = non-fluent, 5 = native-level fluency). Participants rated their overall language ability, and their abilities in speaking and listening in casual conversations, speaking and listening in formal situations, reading, and writing in each language. Final average pre- and post-ABI *LAR* scores were computed for each language using all the above-mentioned ratings. *Daily Use* gathered information on participants' weekday and weekend language use with conversational partners on an hour-by-hour basis. The resulting metric represented a percentage of overall time participants used each language. *Family Proficiency* reflected participants' estimates of their mother's, father's, and siblings' L1 and L2 proficiency on a 0% (not confident) to 100% (strong confident) scale, in 25% increments. Percentages for all family members were averaged, resulting in a single composite *Family Proficiency* score in each language. *Educational History* reported the use of, exposure to, and preference of each language during elementary school, high school, and college, resulting in a percentage of *Educational History* for each language. *Lifetime Exposure* reported the average percentage of time that participants heard, spoke, and read in each language throughout their life. Likewise, *Lifetime Confidence* reflected the participants' self-reported confidence in hearing, speaking, and reading in L1 and L2 throughout their lifetime. For both *Lifetime Exposure* and *Lifetime Confidence*, percentages were initially reported in 3-year intervals from ages 0 to 30 with a final interval for age 30 and up, and with two additional intervals for BPWA (from age 30 until age of ABI onset, and from this timepoint to the date of LUQ administration). Final percentages resulted from averaging across the age intervals and weighting by the participants' age.

All participants completed the LUQ with the help of a trained research assistant as needed. Additionally, for BPWA, caregivers were present to corroborate reported language experience. Language experience metrics are summarized in Table 1. The full datasets are provided in Supplementary Table 2 for HB and Supplementary Table 3 for BPWA.

**Table 1:** Average language use questionnaire metrics for bilingual people with aphasia and healthy bilinguals.

**Group**		**Pre-ABI LAR**		**Post-ABI LAR**		**Pre-ABI use**		**Post-ABI use**		**Family proficiency**		**Lifetime exposure**		**Lifetime confidence**		**Educational history**	
		**L1**	**L2**	**L1**	**L2**	**L1**	**L2**	**L1**	**L2**	**L1**	**L2**	**L1**	**L2**	**L1**	**L2**	**L1**	**L2**
BPWA	*Mean*	0.95	0.82	0.13	0.10	0.54	0.46	0.59	0.41	0.97	0.46	0.61	0.39	0.91	0.58	0.67	0.33
	*SD*	0.13	0.24	0.05	0.05	0.29	0.29	0.32	0.32	0.09	0.33	0.24	0.24	0.17	0.32	0.35	0.35
HB	*Mean*	0.97	0.83	N/A	N/A	0.40	0.60	N/A	N/A	0.94	0.52	0.64	0.36	0.94	0.55	0.74	0.26
	*SD*	0.07	0.17	N/A	N/A	0.31	0.31	N/A	N/A	0.14	0.30	0.19	0.19	0.12	0.28	0.30	0.30

*LAR, language ability rating; BPWA, bilingual people with aphasia; HB, healthy bilingual; L1, first-acquired language; L2, second-acquired language; ABI, acquired brain injury; N/A, no data available*.

## Dataset Overview and Analysis

### Description

The complete LEX-BADAT dataset with raw and processed participant data is available at https://osf.io/jaxsg/?view_only=11d61617d5cf49d39ec9aa446f21c4f7 in a tab-delimited plain text format and as a Microsoft Excel® spreadsheet. This dataset will be updated with the processing of approximately half of each original sample (i.e., update upon each additional 40 BPWA and 15 HB). No individually identifiable information aside from the aforementioned information has been included in these files, and both groups of participants have been coded to preserve their anonymity. The raw data provides individual values of the aforementioned self-reported measures as follows: measures of pre- and post-ABI *Daily Use, Family Proficiency, Educational History, Lifetime Exposure, Lifetime Confidence* are scaled from 0 to 100; pre- and post-ABI *LAR* are scaled from 1 to 5, and *AoA* is the age of L2 learning onset. Correlation matrices of these L1 and L2 raw values for both HB and BPWA are shown in Supplementary Figure 1.

### Data Analysis

Given that the LUQ collects several metrics relating to an individual history of bilingualism resulting in high-dimensional multicollinear data, a PCA approach was implemented to reduce the dimensionality of the LUQ variables while maximally retaining the variance present in the dataset. The statistical analyses were performed using R Statistical Software (R Foundation for Statistical Computing, Vienna, Austria). First, multivariate imputation by chained equations was completed using the “mice” package in R to impute missing values for some BPWA using predictive mean matching. This process was not required for the HB dataset. Next, all input variables for BPWA and HB data were standardized and AoA was reverse-coded (i.e., multiplied by −1 for ease of interpretation) for both groups. Suitability of the data for factor analysis was examined using the “psych” package in R. The Kaiser-Meyer-Olkin index for all four analyses showed suitability for factor analysis (L1 BPWA: 0.84; L2 BPWA: 0.89; L1 HB: 0.73; L2 HB: 0.76), and Bartlett's test of sphericity was significant for all four analyses (L1 BPWA: χ^2^ = 380.78; L2 BPWA: χ^2^ = 3774.00; L1 HB: χ^2^ = 85.06; L2 HB: χ^2^ = 121.65; all *p* < 0.001). Then, separate PCAs were performed on L1 and L2 LUQ metrics for both BPWA and HB using the *principal* function in “psych,” resulting in four sets of solutions. The Kaiser-Guttman criterion (extract components with an eigenvalue >1.0; Yeomans and Golder, 1982) was used to retain components that captured more variance than any single variable. A varimax rotation was applied on the resulting solutions with more than one component to produce orthogonal components and maximize interpretability. Significant loadings were considered those with |loading| ≥ 0.60.

### Principal Component Analysis Output

The PCA results for BPWA revealed a two-component solution in L1, cumulatively explaining 61.81% of the variance in L1 BPWA LUQ data, and a two-component solution in L2, cumulatively explaining 71.32% of the variance in the L2 BPWA LUQ data. Each component was labeled (e.g., *Background*) to be reflective of the items significantly loading onto it. L1 Rotated Component (RC) 1 comprised pre- and post-ABI *Daily Use, Lifetime Exposure*, and *Educational History*, and L1 RC2 consisted of *Family Proficiency* and *Lifetime Confidence*. Notably, for L1 RC2, pre- and post-ABI *LARs* approached the 0.60 cut-off: 0.56 and 0.57, respectively. The component loadings for L2 revealed that L2 RC1 consisted of *AoA, Family Proficiency, Lifetime Exposure, Lifetime Confidence*, and *Educational History*, and L2 RC2 included pre- and post-ABI *LAR*, and pre-ABI *Daily Use*.

Results for HB revealed a one-component solution in L1, explaining 50% of the variance in L1 HB LUQ data, and a two-component solution in L2 cumulatively explaining 72% of the variance L2 HB LUQ data. Using a threshold of 0.60, the component loadings indicated that L1 Principal Component (PC) 1 consisted of *LAR, Educational History, Lifetime Exposure*, and *Lifetime Confidence*. The component loadings for L2 indicated that L2 RC1 was comprised of *AoA, Family Proficiency, Educational History*, and *Lifetime Confidence*, and L2 RC2 included *LAR, Daily Use*, and *Lifetime Exposure*. Retained components and their respective loadings are shown for each group in L1 and L2 in Table 2. Visualizations of loadings are found in Supplementary Figure 2 for HB and Supplementary Figure 3 and BPWA. Individual component loadings for each group in L1 and L2 are found in tab-delimited plain text format and as Microsoft Excel® spreadsheets in the LEX-BADAT dataset.

**Table 2:** Results of the principal component analysis conducted on L1 and L2 for bilingual people with aphasia and healthy bilinguals.

	**PCA on LUQ L1**		**PCA on LUQ L2**	
**BPWA LUQ metrics**	**RC1 (use/background)**	**RC2 (confidence/family proficiency)**	**RC1 (background/confidence)**	**RC2 (ability/use)**
AoA	–	–	**0.78**	0.22
Pre-ABI LAR	0.22	0.56	0.51	**0.61**
Post-ABI LAR	0.23	0.57	0.01	**0.88**
Pre-ABI use	**0.85**	0.04	0.46	**0.64**
Post-ABI Use	**0.86**	0.18	0.59	0.52
Family proficiency	0.06	**0.67**	**0.91**	0.00
Educational history	**0.74**	0.44	**0.74**	0.39
Lifetime exposure	**0.82**	0.36	**0.73**	0.52
Lifetime confidence	0.16	**0.78**	**0.81**	0.37
% Variance	47%	14%	60%	11%
	**PCA on LUQ L1**		**PCA on LUQ L2**	
**HB LUQ metrics**	**PC1 (ability/background/confidence)**		**RC1 (background/confidence)**	**RC2 (ability/use/exposure)**
AoA	–		**0.81**	0.17
LAR	**0.84**		0.21	**0.83**
Use	0.50		−0.05	**0.77**
Family proficiency	0.39		**0.80**	−0.04
Educational history	**0.94**		**0.64**	0.58
Lifetime exposure	**0.81**		0.58	**0.72**
Lifetime confidence	**0.81**		**0.69**	0.58
% Variance	50%		37%	36%

*Component loadings exceeding 0.60 are marked in bold*.

*BPWA, bilingual people with aphasia; HB, healthy bilinguals; L1, first-acquired language; L2, second-acquired language; AoA, L2 age of acquisition; Pre-ABI, pre-acquired brain injury; post-ABI, post-acquired brain injury; LAR, language ability ratings; PCA, principal component analysis; PC, principal component; RC, rotated component*.

## Contextualizing LEX-BADAT

The LEX-BADAT dataset comprises a comprehensive collection of bilingual language experience metrics in Spanish-English HB and BPWA using the LUQ (Kastenbaum et al., 2019). Four PCAs were conducted on LUQ self-reported measures to (i) parsimoniously reduce the dimensionality of the raw LUQ data for ease in statistical analyses using this dataset, and (ii) identify the latent variables underlying the assessment of Spanish-English bilingual language experience. For BPWA, results of the PCA for both L1 and L2 revealed two components in each language with eigenvalues >1.0. For HB, results of the PCA for L1 revealed one component with an eigenvalue >1.0, and the PCA for L2 revealed two components with eigenvalues >1.0.

Given the variability across studies in quantifying and referring to different language experience metrics, the components were interpreted in terms of the information they were likely conveying. Thus, components containing any combination of the following metrics: *AoA, Educational History, Family Proficiency*, and *Lifetime Exposure* were labeled as *Background*. The label *Use* was given to components containing *Daily Use* (pre- and/or post-ABI *Daily Use* for BPWA). The label *Ability* was given to components containing *Language Ability Ratings* (*LARs*; pre- and/or post for BPWA). The label *Confidence* was given to components containing *Lifetime Confidence*, and labels *Family Proficiency* or *Exposure* were given to components that contained *Family Proficiency* or *Lifetime Exposure* independent of other *Background* metrics.

Using the aforementioned labels, the PCA for L1 in BPWA could be separated into two groupings: *Use/Background* and *Confidence/Family Proficiency*. Here, L1 *Use/Background* consisted of pre- and post-ABI *Daily Use, Lifetime Exposure*, and *Educational History*. L1 *Family Proficiency/Confidence* comprised *Family Proficiency* and *Lifetime Confidence*. The PCA for L2 in BPWA could be divided into L2 *Background/Confidence* and L2 *Ability/Use*. L2 *Background/Confidence* consisted of L2 *AoA, Family Proficiency, Lifetime Exposure, Educational History*, and *Lifetime Confidence*, while L2 *Ability/Use* was comprised of pre- and post-ABI *LAR* and pre-ABI *Daily Use*.

For HB, the PCA for L1 consisted of one component called L1 *Ability/Background/Confidence*. This component was comprised of *LAR, Educational History, Lifetime Exposure*, and *Lifetime Confidence*. The PCA for L2 revealed two components: *Background/Confidence* and *Ability/Use/Exposure*. L2 *Background/Confidence* was comprised of L2 *AoA, Family Proficiency, Educational History*, and *Lifetime Confidence*. Finally, L2 Ability/*Use/Exposure* consisted of *LAR, Daily Use*, and *Lifetime Exposure*.

Multivariate techniques, such as common factor analysis and PCA, are commonly applied in aphasia research given the inherent multifactoriality of symptoms secondary to neurological impairment (Gilmore et al., 2019; Meier et al., 2019; Wilson and Hula, 2019).

Previously, Peñaloza et al. (2019) used component scores from a PCA of L1 and L2 LUQ metrics of 27 Spanish-English BPWA (included in LEX-BADAT) to examine the relationship between pre-stroke language proficiency and post-stroke lexical-semantic performance. The authors found that the component scores extracted were significantly predictive of post-stroke lexical-semantic performance in both L1 and L2, both in simple regressions and as an interaction term with the language (i.e., L1 or L2). Further, they revealed that L1 pre-stroke proficiency (in component scores) was a better predictor for BPWA whose L1 was English, whereas L2 pre-stroke proficiency was a better predictor of L2 lexical-semantic performance for BPWA whose L1 was Spanish, possibly reflective of the heterogeneity in language experience and performance secondary to language immersion in the United States among the population.

Furthermore, using similar methods as those described here, Carpenter et al. (2021) found that BPWA language experience in L1 and L2, including *Background, Use*, and *Exposure* in L1, and *Background, Use, Environment*, and *Exposure* in L2, significantly predicted semantic switching performance during verbal fluency tasks, implicating the relationship between pre-ABI language experience and post-ABI language and semantic executive control.

The potential and utility of LEX-BADAT lies on its relatively large sample size including both BPWA and HB across varying profiles of bilingualism and the resulting scores derived from the PCA conducted independently in their L1 and L2. Performing dimensionality reduction is problematic for studies with smaller sample sizes; however, LEX-BADAT may offer a more reliable solution to address important research questions with BPWA using a manageable number of variables representative of their history of bilingualism. For example, Falconer et al. (in preparation), investigated the relationship between language experience measures and translation performance in BPWA using the component scores presented here. Additional component scores for participants not included in the present analyses, obtained by projecting normalized LUQ data onto the previously established PCA space, were found to predict translation performance. Thus, the components reported here can serve as standardized metrics of language experience that provide the opportunity to generate novel predictors, e.g., component scores corresponding to our principal components for participants not originally included in the analyses.

## Conclusion

LEX-BADAT consists of individual-level data from a large group of BPWA and a group of healthy bilingual speakers who completed the LUQ (Kastenbaum et al., 2019) to assess their bilingual language experience. This dataset also provides component scores resulting from a PCA conducted on their L1 and L2 which can be used to inform analyses seeking to examine the influences of language experience on performance on, for example, lexical-semantic tasks (Peñaloza et al., 2019), and lexical access and cognitive control *via* verbal fluency tasks (Carpenter et al., 2021). We hope that in transparently describing our resources and methodology, we improve practices in the quantification of bilingual language experience and subsequent use of terminology (e.g., *Background*) when referring to predictors in statistical analyses employing this set of data.

Finally, similar to other datasets relating to language experience, e.g., BEST (de Bruin et al., 2017), LEX-BADAT provides broad insight into the structure of Spanish-English language experiences of bilinguals in relatively linguistically diverse, primarily American communities, and specifically into the experiences of post-ABI bilinguals experiencing aphasia. In considering post-ABI language experience, our analyses are a holistic view of a critically underserved clinical population, capturing linguistic activity both before and after the onset of a period of chronically disordered lexical access.

## Data Availability Statement

The datasets presented in this study can be found in online repositories. The names of the repository/repositories and accession number(s) can be found in the article/Supplementary Material.

## Ethics Statement

The studies involving human participants were reviewed and approved by Ethical Committee of the University of Texas at Austin and the Ethical Committee of Boston University. The patients/participants provided their written informed consent to participate in this study.

## Author Contributions

MM, EC, and SK contributed to conception of the project and methods of pre- and post-processing of data. MM and EC curated the data and wrote the first draft of the manuscript. MM carried out the statistical approach. MM, EC, IF, and CP contributed to writing the manuscript. All authors contributed toward acquisition of data. All authors contributed to manuscript revision, read, and approved the submitted version.

## Funding

CP was supported by the Juan de la Cierva-Incorporación Program (IJC2018-037818) funded by Ministerio de Ciencia e Innovación, Agencia Estatal de Investigación MCIN/AEI 10.13039/501100011033. This work was supported by the National Institutes of Health/National Institute on Deafness and Other Communication Disorders through grant T32DC013017 awarded to MM and grant U01DC014922 awarded to SK. The content is solely the responsibility of the authors and does not necessarily represent the official views of the National Institutes of Health.

## Conflict of Interest

SK serves as a consultant for Constant Therapy Health with no scientific overlap with the present study. The remaining authors declare that the research was conducted in the absence of any commercial or financial relationships that could be construed as a potential conflict of interest.

## Publisher's Note

All claims expressed in this article are solely those of the authors and do not necessarily represent those of their affiliated organizations, or those of the publisher, the editors and the reviewers. Any product that may be evaluated in this article, or claim that may be made by its manufacturer, is not guaranteed or endorsed by the publisher.
