# Supplementary material for: LEX-BADAT: Language EXperience in Bilinguals With and Without Aphasia DATaset
Source: Front Psychol. 2022 Jun 13;13:875928. doi: 10.3389/fpsyg.2022.875928 (PMC9234733; doi:10.3389/fpsyg.2022.875928)
Supplement: Supplementary file 1 [file Data_Sheet_1.docx]

**Supplementary Materials**

**Supplementary Table 1**: Lesion and clinical information for bilingual people with aphasia.

| **BPWA** | **Age** | **Sex** | **MPO** | **L1** | **AoA** | **Lesion Info** | **L1 WAB AQ** | **L1 Aphasia Subtype** | **L2 WAB AQ** | **L2 Aphasia Subtype** |
| --- | --- | --- | --- | --- | --- | --- | --- | --- | --- | --- |
| P1 | 43 | M | 84 | Span | 19 | Right basal ganglia CVA | N/A | N/A | 71.6 | Anomic |
| P2 | 36 | M | 170 | Span | 6 | TBI | N/A | N/A | 73.6 | Conduction |
| P3 | 77 | F | 4 | Span | 69 | CVA | N/A | N/A | 6 | Global |
| P4 | 65 | F | 5 | Span | 45 | Left MCA CVA involving precentral gyrus | N/A | N/A | N/A | N/A |
| P5 | 45 | M | 83 | Span | 45 | CVA | N/A | N/A | N/A | N/A |
| P6 | 76 | M | 11 | Span | 40 | Infarct in left internal capsule and left parietal lobe | N/A | N/A | N/A | N/A |
| P7 | 33 | F | 3 | Span | 12 | Left MCA CVA | N/A | N/A | 24.9 | Wernicke's |
| P8 | 54 | M | 16 | Span | 12 | CVA involving left inferior cerebellum, left basal ganglia, insula, and frontal, parietal, and temporal lobes | 24.3 | Broca's | 16.7 | Broca's |
| P9 | 46 | M | 1 | Span | 16 | Left CVA involving head of caudate and anterior putamen | N/A | N/A | N/A | N/A |
| P10 | 62 | M | 1 | Span | 20 | CVA | N/A | N/A | N/A | N/A |
| P11 | 82 | M | 411 | Span | 35 | Right posterior parieto-occipital infarct | 55.7 | Broca's | 29.6 | Global |
| P12 | 54 | F | 58 | Span | 6 | Left borderzone infarcts between the ACA and MCA | 74.1 | Anomic | 68.5 | Broca's |
| P13 | 31 | M | 27 | Span | 5 | Hemorrhage in left base of pons | N/A | N/A | N/A | N/A |
| P14 | 29 | M | 42 | Span | 15 | TBI | N/A | N/A | N/A | N/A |
| P15 | 51 | F | N/A | Span | 7 | Left CVA, Right CVA | N/A | N/A | N/A | N/A |
| P16 | 49 | F | 16 | Span | 12 | TBI | N/A | N/A | N/A | N/A |
| P17 | 28 | F | 59 | Eng | 4 | Multiple CVAs secondary to Moyamoya disease | 93.4 | Anomic | 81.4 | Anomic |
| P18 | 44 | M | 28 | Eng | 6 | Left MCA CVA | 89.8 | Anomic | 84.5 | Anomic |
| P19 | 63 | F | 27 | Span | 25 | Left CVA | N/A | N/A | 20.7 | Global |
| P20 | 24 | F | 13 | Span | 5 | Left CVA | 27.3 | Broca's | 37.3 | Broca's |
| P21 | 26 | F | 129 | Span | 5 | Left tumor in posterior centrum semiovale | 77.5 | Anomic | 67.6 | Wernicke’s |
| P22 | 44 | F | 13 | Span | 13 | Left CVA | N/A | N/A | N/A | N/A |
| P23 | 58 | F | 69 | Span | 10 | Left CVA | N/A | N/A | N/A | N/A |
| P24 | 48 | M | 53 | Span | 5 | TBI | N/A | N/A | N/A | N/A |
| P25 | 68 | M | 244 | Span | 27 | Left MCA CVA | 76 | Anomic | 71.3 | Conduction |
| P26 | 47 | F | 53 | Span | 18 | Left MCA temporoparietal infarct | 79.1 | Conduction | 54.4 | Broca's |
| P27 | 53 | M | 37 | Span | 12 | Left CVA | 51.3 | Wernicke’s | 47.5 | Wernicke’s |
| P28 | 77 | M | 26 | Span | 18 | Left MCA CVA involving precentral gyrus | 67.4 | Broca's | 64.7 | Broca's |
| P29 | 77.9 | F | 38 | Span | 10 | Left MCA hemorrhage | 78.9 | Anomic | 76.8 | Conduction |
| P30 | 54 | M | 25 | Span | 6 | Left MCA CVA | N/A | N/A | N/A | N/A |
| P31 | 70 | M | 2 | Span | 15 | Left frontal lobe CVA | 57.3 | Conduction | 39.8 | Broca's |
| P32 | 27 | F | 48 | Span | 11 | Left CVA | 72.3 | Anomic | 66.4 | Broca's |
| P33 | 37 | F | 9 | Span | 6 | Left CVA | N/A | N/A | 69.8 | Anomic |
| P34 | 46 | F | 110 | Span | 9 | Right CVA | N/A | N/A | 92.2 | Anomic |
| P35 | 69 | M | 10 | Eng | 3 | Left CVA | 46.5 | Anomic | 35.9 | Wernicke’s |
| P36 | 62 | M | 23 | Span | 16 | Left MCA CVA | 11.4 | Broca's | 9.5 | Broca's |
| P37 | 47 | F | 12 | Span | 16 | Left anterior MCA infarct | 82.4 | Anomic | 71.2 | Anomic |
| P38 | 56 | M | 51 | Span | 5 | Left CVA | 81.2 | Anomic | 91 | Anomic |
| P39 | 39 | M | 40 | Span | 21 | Left CVA | 21 | Broca's | 39.5 | Broca's |
| P40 | 42 | M | 22 | Eng | 5 | Left CVA | 94.6 | Anomic | 57.8 | Broca's |
| P41 | 62 | M | 52 | Eng | 8 | Left CVA | 85.2 | Anomic | 78.6 | Anomic |
| P42 | 66 | F | 5 | Span | 17 | Left CVA | N/A | N/A | N/A | N/A |
| P43 | 68 | F | 79 | Span | 21 | Left MCA CVA | 47.5 | Broca's | 45.5 | Broca's |
| P44 | 81 | F | 163 | Span | 42 | Left frontal and parietal CVAs; right occipital and cerebellar CVAs | 75 | Anomic | 65.6 | Anomic |
| P45 | 40 | M | 7 | Span | 30 | Left basal ganglia CVA | N/A | N/A | N/A | N/A |
| P46 | 66 | F | 11 | Span | 8 | Left CVA, Hx of right CVA | 83.2 | Anomic | 64.2 | Anomic |
| P47 | 55 | M | 46 | Span | 5 | Left MCA CVA | 92.6 | Anomic | 97.2 | Anomic |
| P48 | 67 | F | 8 | Span | 21 | Left MCA CVA; bilateral cerebellar CVAs | 21.7 | Global | 23.9 | Global |
| P49 | 50 | F | 31 | Span | 16 | Left temporoparietal CVA | 92.6 | Anomic | 69.8 | Wernicke’s |
| P50 | 65 | F | 78 | Span | 7 | Left CVA | N/A | N/A | 32.6 | Broca's |
| P51 | 53 | M | 6 | Span | 9 | Left CVA | 41.4 | Broca's | 40.1 | Broca's |
| P52 | 58 | M | 8 | Span | 17 | Left frontoparietal CVA; left basal ganglia CVA | 14.7 | Broca's | 7.8 | Global |
| P53 | 38 | F | 6 | Span | 8 | Left CVA | 68.3 | Anomic | 82.6 | Anomic |
| P54 | 59 | M | 29 | Span | 5 | Left MCA CVA | 21.7 | Broca's | 50.9 | Broca's |
| P55 | 49 | F | 17 | Span | 3 | Left CVA | N/A | N/A | N/A | N/A |
| P56 | 32 | M | 9 | Span | 24 | Left basal ganglia CVA | 76.1 | Anomic | 20.5 | Global |
| P57 | 56 | F | 69 | Span | 1 | Left frontal and basal ganglia CVA | 60.5 | Anomic | 89.6 | Anomic |
| P58 | 62 | M | 9 | Span | 35 | Left MCA CVA | 85.4 | Anomic | 61.7 | Wernicke’s |
| P59 | 47 | M | 9 | Span | 5 | CVA | N/A | N/A | N/A | N/A |
| P60 | 59 | M | 8 | Span | 0 | Left CVA | 15.8 | Broca's | 10.6 | Broca's |
| P61 | 18 | M | 13 | Span | 3 | Left frontal lobe CVA | 27 | Broca's | 72.6 | Anomic |
| P62 | 73 | F | 64 | Span | 5 | Left CVA | 92.4 | Anomic | 93.8 | Anomic |
| P63 | 57 | M | 7 | Span | 27 | Left CVA | 19.6 | Broca's | 14.7 | Broca's |
| P64 | 37 | F | 20 | Span | 8 | Left MCA CVA | 90.2 | Anomic | 84 | Anomic |
| P65 | 65 | M | 6 | Span | 32 | Left basal ganglia CVA | N/A | N/A | N/A | N/A |
| P66 | 64 | N/A | N/A | Span | 21 | Left CVA | N/A | N/A | N/A | N/A |
| P67 | 58 | N/A | N/A | Span | 5 | Left CVA | N/A | N/A | N/A | N/A |
| P68 | 53 | N/A | N/A | Span | 3 | Left CVA | N/A | N/A | N/A | N/A |
| P69 | 53 | N/A | N/A | Span | 6 | Left CVA | N/A | N/A | N/A | N/A |
| P70 | 73 | N/A | N/A | Span | 17 | Left CVA | N/A | N/A | N/A | N/A |
| P71 | 75 | N/A | N/A | Span | 28 | Left CVA | N/A | N/A | N/A | N/A |
| P72 | 85 | N/A | N/A | Span | 69 | Left CVA | N/A | N/A | N/A | N/A |
| P73 | 88 | N/A | N/A | Span | 5 | Left CVA | N/A | N/A | N/A | N/A |
| P74 | 41 | N/A | N/A | Span | 18 | Left CVA | N/A | N/A | N/A | N/A |
| P75 | 41 | N/A | N/A | Span | 9 | Left CVA | N/A | N/A | N/A | N/A |
| P76 | 33 | M | 12 | Eng | 11 | Left CVA | N/A | N/A | N/A | N/A |
| P77 | 58 | M | 56 | Span | 0 | Left MCA CVA | 4.7 | Global | 5.5 | Global |
| P78 | 53 | F | 44 | Eng | 3 | Left MCA and PCA CVAs | 90 | Anomic | 68.8 | Anomic |
| P79 | 54 | F | 28 | Eng | 12 | Left CVA | 96.5 | Anomic | 60.8 | Broca's |
| P80 | 29 | F | 105 | Eng | 14 | Left MCA CVA | N/A | N/A | N/A | N/A |
| P81 | 21 | F | 23 | Span | 3 | Left CVA, right occipital and right cerebellar infarcts | 34.4 | Broca's | 53.3 | Wernicke’s |
| P82 | 63 | M | 385 | Eng | 3 | Left CVA | 47.7 | Broca's | 15.4 | Broca's |
| P83 | 44 | F | 34 | Eng | 0 | Left MCA CVA | N/A | N/A | 36.5 | Broca's |
| P84 | 52 | F | 32 | Eng | 13 | Left frontoparietal hemorrhage | 97.3 | Anomic | N/A | N/A |
| P85 | 59 | N/A | N/A | Eng | 0 | Left CVA | N/A | N/A | N/A | N/A |

Note: Aphasia subtypes are based on WAB-R (Kertesz, 2006) classifications. L1 WAB-R AQ scores are not significantly different from L2 WAB-R AQ scores (*p* = .568). BPWA = bilingual people with aphasia; MPO = months post-onset; L1 = first-acquired language; L2 = second-acquired language; ACA = anterior cerebral artery; MCA = middle cerebral artery; PCA = posterior cerebral artery; CVA = cerebrovascular accident; WAB = Western Aphasia Battery; AQ = Aphasia Quotient; M = male; F = female; N/A = no data available.

**Supplementary Table 2.** Language Use Questionnaire metrics for healthy bilinguals.

|  |  |  | **L1** | | | | | |  | **L2** | | | | | | |
| --- | --- | --- | --- | --- | --- | --- | --- | --- | --- | --- | --- | --- | --- | --- | --- | --- |
| **HB** | **Sex** | **L1** | **Use^a^** | **Expo.^a^** | **Edu.^a^** | **Conf.^b^** | **LAR^c^** | **Fam.^c^** |  | **AoA** | **Use^a^** | **Expo.^a^** | **Edu.^a^** | **Conf.^b^** | **LAR^c^** | **Fam.^c^** |
| P1 | M | Span | 0.03 | 0.39 | 0.22 | 0.49 | 0.80 | 1.00 |  | 0 | 0.97 | 0.61 | 0.78 | 0.89 | 1.00 | 0.63 |
| P2 | M | Span | 0.42 | 0.64 | 0.72 | 0.96 | 0.91 | 0.75 |  | 20 | 0.58 | 0.36 | 0.28 | 0.41 | 0.89 | 0.63 |
| P3 | F | Span | 0.28 | 0.62 | 0.61 | 0.79 | 1.00 | 1.00 |  | 4 | 0.72 | 0.38 | 0.39 | 0.55 | 0.74 | 0.92 |
| P4 | F | Span | 0.17 | 0.71 | 0.89 | 1.00 | 1.00 | 1.00 |  | 28 | 0.83 | 0.29 | 0.11 | 0.40 | 0.80 | 0.33 |
| P5 | F | Span | 0.47 | 0.70 | 0.83 | 1.00 | 1.00 | 1.00 |  | 26 | 0.53 | 0.30 | 0.17 | 0.34 | 1.00 | 0.25 |
| P6 | M | Span | 0.00 | 0.60 | 0.94 | 1.00 | 1.00 | 1.00 |  | 12 | 1.00 | 0.40 | 0.06 | 0.57 | 0.91 | 0.25 |
| P7 | F | Span | 0.28 | 0.76 | 1.00 | 1.00 | 1.00 | 1.00 |  | 7 | 0.72 | 0.24 | 0.00 | 0.75 | 0.80 | 0.33 |
| P8 | M | Span | 0.09 | 0.55 | 0.83 | 1.00 | 1.00 | 1.00 |  | 15 | 0.91 | 0.45 | 0.17 | 0.72 | 0.86 | 0.25 |
| P9 | F | Span | 0.22 | 0.80 | 1.00 | 1.00 | 1.00 | 0.42 |  | 36 | 0.78 | 0.20 | 0.00 | 0.18 | 0.97 | 0.92 |
| P10 | M | Span | 0.21 | 0.68 | 0.89 | 0.94 | 1.00 | 1.00 |  | 7 | 0.79 | 0.32 | 0.11 | 0.55 | 0.91 | 0.25 |
| P11 | F | Span | 0.19 | 0.39 | 0.11 | 0.76 | 0.80 | 0.92 |  | 6 | 0.81 | 0.61 | 0.89 | 0.61 | 1.00 | 0.67 |
| P12 | F | Span | 0.22 | 0.37 | 0.00 | 0.69 | 0.86 | 0.50 |  | 0 | 0.78 | 0.63 | 1.00 | 0.99 | 1.00 | 0.92 |
| P13 | F | Span | 0.82 | 0.81 | 0.78 | 1.00 | 1.00 | 1.00 |  | 7 | 0.18 | 0.19 | 0.22 | 0.47 | 0.89 | 0.42 |
| P14 | F | Span | 0.45 | 0.44 | 0.28 | 0.86 | 0.97 | 0.75 |  | 0 | 0.55 | 0.56 | 0.72 | 1.00 | 1.00 | 1.00 |
| P15 | F | Span | 0.97 | 0.89 | 1.00 | 1.00 | 1.00 | 1.00 |  | 7 | 0.03 | 0.11 | 0.00 | 0.15 | 0.43 | 0.58 |
| P16 | F | Span | 1.00 | 0.89 | 1.00 | 1.00 | 1.00 | 1.00 |  | 40 | 0.00 | 0.11 | 0.00 | 0.00 | 0.49 | 0.33 |
| P17 | F | Span | 0.38 | 0.45 | 0.33 | 0.94 | 0.80 | 1.00 |  | 5 | 0.63 | 0.55 | 0.67 | 0.98 | 1.00 | 0.92 |
| P18 | F | Span | 0.07 | 0.64 | 0.78 | 1.00 | 1.00 | 1.00 |  | 21 | 0.93 | 0.36 | 0.22 | 0.37 | 0.86 | 0.13 |
| P19 | F | Span | 0.17 | 0.51 | 0.67 | 1.00 | 1.00 | 1.00 |  | 18 | 0.83 | 0.49 | 0.33 | 0.69 | 1.00 | 0.67 |
| P20 | F | Span | 0.73 | 0.65 | 0.94 | 0.93 | 1.00 | 1.00 |  | 26 | 0.28 | 0.35 | 0.06 | 0.48 | 0.97 | 1.00 |
| P21 | F | Span | 0.07 | 0.26 | 0.28 | 1.00 | 1.00 | 1.00 |  | 12 | 0.93 | 0.74 | 0.72 | 0.77 | 1.00 | 0.42 |
| P22 | F | Span | 0.61 | 0.29 | 0.67 | 1.00 | 1.00 | 1.00 |  | 3 | 0.39 | 0.71 | 0.33 | 0.89 | 1.00 | 0.42 |
| P23 | F | Span | 0.72 | 0.72 | 1.00 | 1.00 | 1.00 | 1.00 |  | 22 | 0.28 | 0.28 | 0.00 | 0.32 | 0.77 | 0.17 |
| P24 | M | Span | 0.86 | 0.74 | 1.00 | 1.00 | 1.00 | 1.00 |  | 27 | 0.14 | 0.26 | 0.00 | 0.26 | 0.60 | 0.33 |
| P25 | F | Span | 0.96 | 0.69 | 0.89 | 1.00 | 1.00 | 1.00 |  | 3 | 0.04 | 0.31 | 0.11 | 0.80 | 0.74 | 0.58 |
| P26 | F | Span | 0.57 | 1.00 | 0.89 | 1.00 | 1.00 | 1.00 |  | 38 | 0.43 | 0.00 | 0.11 | 0.00 | 0.69 | 0.00 |
| P27 | F | Span | 0.13 | 0.63 | 0.94 | 0.93 | 0.97 | 1.00 |  | 6 | 0.87 | 0.37 | 0.06 | 0.62 | 0.77 | 0.50 |
| P28 | F | Eng | 0.05 | 0.81 | 0.94 | 1.00 | 1.00 | 1.00 |  | 12 | 0.95 | 0.19 | 0.06 | 0.48 | 0.57 | 0.50 |
| P29 | F | Eng | 0.36 | 0.47 | 0.56 | 0.98 | 0.86 | 0.88 |  | 0 | 0.64 | 0.53 | 0.44 | 0.82 | 0.80 | 1.00 |
| P30 | F | Eng | 0.50 | 0.81 | 0.94 | 0.98 | 1.00 | 1.00 |  | 19 | 0.50 | 0.19 | 0.06 | 0.67 | 0.83 | 0.13 |
| P31 | M | Eng | 0.25 | 0.82 | 0.94 | 1.00 | 1.00 | 1.00 |  | 0 | 0.75 | 0.18 | 0.06 | 0.24 | 0.51 | 0.75 |

Scores are expressed as proportions of ^a^time (for exposure, pre- and post-use, and educational history in L1 and L2), ^b^confidence in L1 and L2, and ^c^family and self-rated proficiency in L1 and L2.

HB = healthy bilinguals; F = female; M = male; L1 = first-acquired language; L2 = second-acquired language; Span = Spanish; Eng = English; AoA = L2 age of acquisition (expressed in years); LAR = language ability rating; Fam. = family proficiency; Edu. = education; Env. = environment; Expo. = exposure; Conf. = confidence.

**Supplementary Table 3.** Language Use Questionnaire metrics for bilingual people with aphasia.

|  |  |  |  | **L1** | | | | | | | | **L2** | | | | | | | |
| --- | --- | --- | --- | --- | --- | --- | --- | --- | --- | --- | --- | --- | --- | --- | --- | --- | --- | --- | --- |
| **BPWA** |  | **L1** | **AoA** | **Pre LAR** | **Post LAR** | **Pre Use** | **Post Use** | **Family Prof.** | **Edu.** | **Expo.** | **Conf.** | **Pre LAR** | **Post LAR** | **Pre Use** | **Post Use** | **Family Prof.** | **Edu.** | **Expo.** | **Conf.** |
|  | **Sex** |  |  |  |  |  |  |  |  |  |  |  |  |  |  |  |  |  |  |
| P1 | M | Span | 19 | 1.00 | 0.12 | 0.78 | 0.78 | 1.00 | 1.00 | 0.72 | 1.00 | 1.00 | 0.04 | 0.22 | 0.22 | 0.33 | 0.00 | 0.28 | 0.40 |
| P2 | M | Span | 6 | 0.40 | 0.04 | 0.34 | 0.34 | 1.00 | 0.00 | 0.26 | 1.00 | 1.00 | 0.08 | 0.66 | 0.66 | 0.67 | 1.00 | 0.74 | 0.81 |
| P3 | F | Span | 69* | 1.00 | 0.12 | 0.92 | 0.92 | 1.00 | 1.00 | 0.85 | 1.00 | 0.20 | 0.04 | 0.08 | 0.08 | 0.00 | 0.00 | 0.15 | 0.00* |
| P4 | F | Span | 45* | 1.00 | 0.16 | 0.98 | 0.98 | 1.00 | 1.00 | 0.87 | 1.00 | 0.40 | 0.08 | 0.02 | 0.02 | 0.00 | 0.00 | 0.13 | 0.13 |
| P5 | M | Span | 45 | 1.00 | 0.16 | 0.46* | 1.00 | 1.00 | 1.00 | 0.94 | 1.00 | 0.80 | 0.04 | 0.54* | 0.00 | 0.00 | 0.00 | 0.06 | 0.06 |
| P6 | M | Span | 40 | 1.00 | 0.20 | 1.00 | 1.00 | 1.00 | 1.00 | 1.00 | 1.00 | 0.10* | 0.04 | 0.00 | 0.00 | 0.00 | 0.00 | 0.00 | 0.15 |
| P7 | F | Span | 12 | 1.00 | 0.08 | 0.54 | 0.54 | 0.92 | 0.72 | 0.71 | 1.00 | 0.80 | 0.04 | 0.46 | 0.46 | 0.67 | 0.28 | 0.29 | 0.52 |
| P8 | M | Span | 12 | 1.00 | 0.08 | 0.78 | 0.90 | 1.00 | 0.92 | 0.73 | 1.00 | 0.60 | 0.08 | 0.22 | 0.10 | 0.08 | 0.08 | 0.27 | 0.21 |
| P9 | M | Span | 16 | 1.00 | 0.12 | 0.49 | 1.00 | 1.00 | 1.00 | 0.62 | 1.00 | 1.00 | 0.04 | 0.51 | 0.00 | 0.00 | 0.00 | 0.38 | 0.49 |
| P10 | M | Span | 20 | 1.00 | 0.08 | 0.77 | 1.00 | 1.00 | 1.00 | 0.44 | 1.00 | 0.80 | 0.04 | 0.23 | 0.00 | 0.01* | 0.00 | 0.56 | 0.00 |
| P11 | M | Span | 35 | 1.00 | 0.20 | 0.62 | 1.00* | 1.00 | 0.89 | 0.80 | 1.00 | 0.80 | 0.12 | 0.38 | 0.00* | 0.17 | 0.11 | 0.20 | 0.36 |
| P12 | F | Span | 6 | 1.00* | 0.16 | 0.55 | 0.10 | 1.00 | 0.89 | 0.67 | 1.00 | 0.40 | 0.04 | 0.46 | 0.90 | 0.67 | 0.11 | 0.33 | 0.28 |
| P13 | M | Span | 5 | 0.80 | 0.12 | 0.18 | 0.15 | 0.67 | 0.06 | 0.29 | 0.68 | 1.00 | 0.16 | 0.82 | 0.85 | 1.00 | 0.94 | 0.71 | 0.93 |
| P14 | M | Span | 15 | 1.00 | 0.20 | 0.93 | 0.96 | 1.00 | 0.75 | 0.97 | 1.00 | 0.80* | 0.12 | 0.07 | 0.04 | 0.33 | 0.25 | 0.03 | 0.17 |
| P15 | F | Span | 7 | 0.80 | 0.14 | 0.31 | 0.50 | 1.00 | 0.25 | 0.23 | 0.20 | 1.00 | 0.16 | 0.69 | 0.50 | 0.67 | 0.75 | 0.77 | 0.96 |
| P16 | F | Span | 12 | 1.00 | 0.20 | 0.18 | 0.22 | 1.00 | 0.78 | 0.59 | 1.00 | 1.00 | 0.20 | 0.82 | 0.78 | 0.17 | 0.22 | 0.41 | 0.46 |
| P17 | F | Eng | 4 | 1.00 | 0.08 | 0.47 | 0.27 | 1.00 | 0.11 | 0.37 | 0.69 | 1.00 | 0.20 | 0.53 | 0.73 | 0.50 | 0.89 | 0.63 | 1.00 |
| P18 | M | Eng | 6 | 1.00 | 0.12 | 0.01 | 0.02 | 1.00 | 0.50 | 0.26 | 0.65 | 0.80 | 0.12 | 0.99 | 0.98 | 1.00 | 0.50 | 0.74 | 0.95 |
| P19 | F | Span | 25 | 0.60 | 0.08 | 0.73 | 0.78 | 1.00 | 1.00 | 0.50 | 0.50 | 0.60 | 0.08 | 0.27 | 0.22 | 0.42 | 0.00 | 0.50 | 0.50 |
| P20 | F | Span | 5 | 1.00 | 0.04 | 0.14 | 0.42 | 0.75 | 0.28 | 0.47 | 0.66 | 1.00 | 0.04 | 0.86 | 0.58 | 0.58 | 0.72 | 0.53 | 0.68 |
| P21 | F | Span | 5 | 0.80 | 0.16 | 0.91 | 0.66 | 1.00 | 0.39 | 0.53 | 0.72 | 0.80 | 0.12 | 0.09 | 0.34 | 0.75 | 0.61 | 0.47 | 0.62 |
| P22 | F | Span | 13 | 1.00 | 0.08 | 0.42 | 0.75 | 1.00 | 0.89 | 0.70 | 1.00 | 1.00 | 0.04 | 0.58 | 0.25 | 0.33 | 0.11 | 0.30 | 0.27 |
| P23 | F | Span | 10 | 0.70 | 0.16 | 0.13 | 0.23* | 1.00 | 0.50 | 0.33 | 0.90 | 1.00 | 0.16 | 0.87 | 0.77* | 1.00 | 0.50 | 0.67 | 0.26 |
| P24 | M | Span | 5 | 0.70 | 0.12 | 0.35 | 0.65 | 0.92 | 0.17 | 0.27 | 0.79 | 1.00 | 0.16 | 0.65 | 0.35 | 0.42 | 0.83 | 0.73 | 0.92 |
| P25 | M | Span | 27 | 0.80 | 0.20 | 0.29 | 0.70 | 1.00 | 0.83 | 0.62 | 1.00 | 1.00 | 0.12 | 0.71 | 0.30 | 0.25 | 0.17 | 0.38 | 0.47 |
| P26 | F | Span | 18 | 1.00 | 0.16 | 0.09 | 0.65 | 1.00 | 1.00 | 0.60 | 1.00 | 0.80 | 0.08 | 0.91 | 0.35 | 0.17 | 0.00 | 0.40 | 0.45 |
| P27 | M | Span | 12 | 1.00 | 0.20 | 0.02 | 0.31 | 1.00 | 1.00 | 0.68 | 1.00 | 1.00 | 0.16 | 0.98 | 0.69 | 0.08 | 0.00 | 0.32 | 0.46 |
| P28 | M | Span | 18 | 1.00 | 0.16 | 0.24 | 0.41 | 1.00 | 0.83 | 0.43 | 1.00 | 1.00 | 0.16 | 0.76 | 0.59 | 0.42 | 0.17 | 0.57 | 0.58 |
| P29 | F | Span | 10 | 1.00 | 0.12 | 0.45 | 0.29 | 1.00 | 0.58 | 0.55 | 1.00 | 1.00 | 0.12 | 0.55 | 0.71 | 0.25 | 0.42 | 0.45 | 0.56 |
| P30 | M | Span | 6 | 1.00* | 0.20 | 1.00 | 0.97 | 1.00 | 0.92 | 0.91 | 1.00* | 0.90* | 0.12 | 0.00 | 0.03 | 0.00 | 0.08 | 0.09 | 0.47* |
| P31 | M | Span | 15 | 1.00 | 0.20 | 0.70 | 0.75 | 1.00 | 0.75 | 0.81 | 1.00 | 1.00 | 0.12 | 0.30 | 0.25 | 0.00 | 0.25 | 0.19 | 0.48 |
| P32 | F | Span | 11 | 1.00 | 0.12 | 0.37 | 0.81 | 1.00 | 0.56 | 0.63 | 0.97 | 1.00 | 0.08 | 0.63 | 0.19 | 0.75 | 0.44 | 0.37 | 0.47 |
| P33 | F | Span | 6 | 1.00 | 0.12 | 0.62 | 0.75 | 1.00 | 0.33 | 0.49 | 1.00 | 0.60 | 0.08 | 0.38 | 0.25 | 0.67 | 0.67 | 0.51 | 0.52 |
| P34 | F | Span | 9 | 0.60 | 0.12 | 0.07 | 0.02 | 0.67 | 0.00 | 0.35 | 0.80 | 1.00 | 0.20 | 0.93 | 0.98 | 1.00 | 1.00 | 0.65 | 0.76 |
| P35 | M | Eng | 3 | 1.00 | 0.08 | 0.25 | 0.06 | 0.92 | 0.00 | 0.50 | 0.81 | 1.00 | 0.08 | 0.75 | 0.94 | 0.83 | 1.00 | 0.50 | 0.98 |
| P36 | M | Span | 16 | 1.00 | 0.16 | 0.66 | 0.50 | 0.92 | 0.83 | 0.67 | 1.00 | 1.00 | 0.16 | 0.34 | 0.50 | 0.13 | 0.17 | 0.33 | 0.42 |
| P37 | F | Span | 16 | 1.00 | 0.16 | 0.60 | 0.52 | 1.00 | 0.92 | 0.69 | 1.00 | 0.80 | 0.16 | 0.40 | 0.48 | 0.42 | 0.08 | 0.31 | 0.48 |
| P38 | M | Span | 5 | 1.00 | 0.20 | 0.75 | 0.32 | 1.00 | 0.75 | 0.31 | 0.91 | 0.80 | 0.16 | 0.25 | 0.68 | 0.42 | 0.25 | 0.69 | 0.93 |
| P39 | M | Span | 21 | 1.00 | 0.08 | 0.35 | 0.54 | 1.00 | 0.94 | 0.76 | 0.99 | 1.00 | 0.08 | 0.65 | 0.46 | 0.17 | 0.06 | 0.24 | 0.44 |
| P40 | M | Eng | 5 | 1.00 | 0.04 | 0.19 | 0.00 | 1.00 | 0.17 | 0.15 | 1.00 | 1.00 | 0.08 | 0.81 | 1.00 | 0.92 | 0.83 | 0.85 | 0.88 |
| P41 | M | Eng | 8 | 0.80 | 0.16 | 0.26 | 0.04 | 1.00 | 0.50 | 0.33 | 1.00 | 1.00 | 0.12 | 0.74 | 0.96 | 0.75 | 0.50 | 0.67 | 0.92 |
| P42 | F | Span | 17 | 1.00 | 0.12 | 0.48 | 1.00* | 1.00 | 0.50 | 0.77 | 0.80 | 1.00 | 0.10 | 0.52 | 0.00* | 0.42 | 0.50 | 0.23 | 0.92 |
| P43 | F | Span | 21 | 1.00 | 0.12 | 0.41 | 0.43 | 1.00 | 0.83 | 0.64 | 1.00 | 1.00 | 0.12 | 0.59 | 0.57 | 0.08 | 0.17 | 0.36 | 0.35 |
| P44 | F | Span | 42 | 0.60 | 0.12 | 1.00 | 0.74 | 1.00 | 1.00 | 1.00 | 1.00 | 0.20 | 0.04 | 0.00 | 0.26 | 0.00 | 0.00 | 0.00 | 0.00 |
| P45 | M | Span | 30 | 1.00 | 0.12 | 0.77 | 0.94 | 1.00 | 1.00 | 0.85 | 1.00 | 0.60 | 0.08 | 0.23 | 0.06 | 0.00 | 0.00 | 0.15 | 0.15 |
| P46 | F | Span | 8 | 1.00 | 0.16 | 0.69 | 0.84 | 1.00 | 0.89 | 0.85 | 1.00 | 0.60 | 0.08 | 0.31 | 0.16 | 0.58 | 0.11 | 0.15 | 0.52 |
| P47 | M | Span | 5 | 0.80 | 0.12 | 0.29 | 0.07 | 0.83 | 0.22 | 0.10 | 0.74 | 0.80 | 0.16 | 0.71 | 0.93 | 0.92 | 0.78 | 0.90 | 0.96 |
| P48 | F | Span | 21 | 1.00 | 0.12 | 0.77 | 0.39 | 1.00 | 1.00 | 0.83 | 1.00 | 0.90 | 0.08 | 0.23 | 0.61 | 0.42 | 0.00 | 0.17 | 0.49 |
| P49 | F | Span | 16 | 1.00 | 0.12 | 0.47 | 0.99 | 1.00 | 1.00 | 0.74 | 0.90 | 0.80 | 0.04 | 0.53 | 0.01 | 0.63 | 0.00 | 0.26 | 0.47 |
| P50 | F | Span | 7 | 1.00 | 0.20 | 0.38 | 1.00 | 1.00 | 0.56 | 0.39 | 1.00 | 1.00 | 0.08 | 0.62 | 0.00 | 1.00 | 0.44 | 0.61 | 0.86 |
| P51 | M | Span | 9 | 1.00 | 0.20 | 0.05 | 0.50 | 1.00 | 0.25 | 0.57 | 1.00 | 1.00 | 0.20 | 0.95 | 0.50 | 0.50 | 0.75 | 0.43 | 0.89 |
| P52 | M | Span | 17 | 1.00 | 0.12 | 0.96 | 1.00 | 1.00 | 1.00 | 0.84 | 1.00 | 0.80 | 0.08 | 0.04 | 0.00 | 0.33 | 0.00 | 0.16 | 0.50 |
| P53 | F | Span | 8 | 1.00 | 0.12 | 0.26 | 0.10 | 1.00 | 0.00 | 0.55 | 1.00 | 1.00 | 0.12 | 0.74 | 0.90 | 0.42 | 1.00 | 0.45 | 0.78 |
| P54 | M | Span | 5 | 1.00 | 0.12 | 0.50 | 0.23 | 1.00 | 0.33 | 0.52 | 1.00 | 1.00 | 0.12 | 0.50 | 0.77 | 0.63 | 0.67 | 0.48 | 0.97 |
| P55 | F | Span | 3 | 1.00 | 0.04 | 0.52 | 0.07 | 1.00 | 0.17 | 0.35 | 0.67 | 1.00 | 0.04 | 0.48 | 0.93 | 0.67 | 0.83 | 0.65 | 0.98 |
| P56 | M | Span | 24 | 1.00 | 0.16 | 0.82 | 0.89 | 1.00 | 1.00 | 0.98 | 1.00 | 0.20 | 0.04 | 0.18 | 0.11 | 0.00 | 0.00 | 0.02 | 0.05 |
| P57 | F | Span | 1 | 0.60 | 0.04 | 0.14 | 0.32 | 1.00 | 0.28 | 0.15 | 0.43 | 1.00 | 0.16 | 0.86 | 0.68 | 0.67 | 0.72 | 0.85 | 0.96 |
| P58 | M | Span | 35 | 1.00 | 0.12 | 0.87 | 0.98 | 1.00 | 1.00 | 0.98 | 1.00 | 0.60 | 0.12 | 0.13 | 0.02 | 0.33 | 0.00 | 0.02 | 0.13 |
| P59 | M | Span | 5 | 0.80 | 0.12 | 0.46 | 0.35 | 0.83 | 0.50 | 0.46 | 0.67 | 0.60 | 0.12 | 0.54 | 0.65 | 0.42 | 0.50 | 0.54 | 0.71 |
| P60 | M | Span | 0 | 1.00 | 0.08 | 0.35 | 0.25 | 1.00 | 0.50 | 0.45 | 1.00 | 1.00 | 0.08 | 0.65 | 0.75 | 0.75 | 0.50 | 0.55 | 0.87 |
| P61 | M | Span | 3 | 1.00 | 0.04 | 0.36 | 0.07 | 0.75 | 0.00 | 0.29 | 0.69 | 1.00 | 0.08 | 0.64 | 0.93 | 0.83 | 1.00 | 0.71 | 0.91 |
| P62 | F | Span | 5 | 1.00 | 0.12 | 0.74 | 0.60 | 1.00 | 0.28 | 0.35 | 0.99 | 1.00 | 0.16 | 0.26 | 0.40 | 0.08 | 0.72 | 0.65 | 0.94 |
| P63 | M | Span | 27 | 1.00 | 0.12 | 0.65 | 0.79 | 0.83 | 0.89 | 0.77 | 1.00 | 0.60 | 0.08 | 0.35 | 0.21 | 0.25 | 0.11 | 0.23 | 0.22 |
| P64 | F | Span | 8 | 1.00 | 0.20 | 0.45 | 0.28 | 1.00 | 1.00 | 0.46 | 0.97 | 1.00 | 0.16 | 0.55 | 0.72 | 0.17 | 0.00 | 0.54 | 0.93 |
| P65 | M | Span | 32 | 1.00 | 0.12 | 0.71 | 1.00 | 1.00 | 1.00 | 1.00 | 1.00 | 0.60 | 0.08 | 0.29 | 0.00 | 0.00 | 0.00 | 0.00 | 0.00 |
| P66 | N/A | Span | 21 | 1.00 | 0.16 | 0.21 | 0.50 | 1.00 | 1.00 | 0.55 | 1.00 | 1.00 | 0.12 | 0.79 | 0.50 | 0.25 | 0.00 | 0.45 | 0.67 |
| P67 | N/A | Span | 5 | 0.60* | 0.08 | 0.42 | 0.42 | 0.92 | 0.22 | 0.37 | 0.59 | 1.00* | 0.12 | 0.58 | 0.58 | 0.83 | 0.78 | 0.63 | 0.78 |
| P68 | N/A | Span | 3 | 0.80 | 0.14 | 0.46 | 0.46 | 1.00 | 0.33 | 0.38 | 0.94 | 1.00 | 0.16 | 0.54 | 0.54 | 1.00 | 0.67 | 0.62 | 0.99 |
| P69 | N/A | Span | 6 | 1.00 | 0.04 | 0.45 | 0.45 | 1.00 | 0.42 | 0.34 | 0.66 | 1.00 | 0.08 | 0.55 | 0.55 | 0.67 | 0.58 | 0.66 | 0.96 |
| P70 | N/A | Span | 17 | 1.00 | 0.16 | 1.00 | 1.00 | 1.00 | 0.75 | 0.66 | 1.00 | 0.90 | 0.08 | 0.00 | 0.00 | 0.88 | 0.25 | 0.34 | 0.83 |
| P71 | N/A | Span | 28 | 1.00 | 0.20 | 0.84 | 0.84 | 1.00 | 1.00 | 0.91* | 0.51 | 0.10 | 0.04 | 0.16 | 0.16 | 0.08 | 0.00 | 0.09* | 0.08 |
| P72 | N/A | Span | 69 | 1.00* | 0.12 | 0.70 | 0.70 | 1.00 | 1.00 | 0.95 | 1.00 | 0.40* | 0.04 | 0.30 | 0.30 | 0.00 | 0.00 | 0.05 | 0.00 |
| P73 | N/A | Span | 5 | 1.00* | 0.04 | 0.01 | 0.01 | 1.00 | 0.00 | 0.29 | 1.00 | 1.00* | 0.08 | 0.99 | 0.99 | 1.00 | 1.00 | 0.71 | 1.00 |
| P74 | N/A | Span | 18 | 1.00 | 0.20 | 0.71 | 0.71 | 1.00 | 1.00 | 0.90 | 1.00 | 0.40 | 0.04 | 0.29 | 0.29 | 0.17 | 0.00 | 0.10 | 0.11 |
| P75 | N/A | Span | 9 | 1.00 | 0.16 | 0.71 | 0.71 | 1.00 | 0.78 | 0.68 | 1.00 | 0.80 | 0.10 | 0.29 | 0.29 | 0.33 | 0.22 | 0.32 | 0.41 |
| P76 | M | Eng | 11 | 1.00 | 0.20 | 0.72 | 1.00 | 1.00 | 1.00 | 0.91 | 1.00 | 0.80 | 0.16 | 0.28 | 0.00 | 0.25 | 0.00 | 0.09 | 0.50 |
| P77 | M | Span | 0 | 1.00 | 0.04 | 0.44 | 0.40 | 1.00 | 0.22 | 0.41 | 1.00 | 1.00 | 0.04 | 0.56 | 0.60 | 0.92 | 0.78 | 0.59 | 1.00 |
| P78 | F | Eng | 3 | 1.00 | 0.20 | 0.74 | 0.98 | 1.00 | 0.94 | 0.59 | 1.00 | 0.80 | 0.12 | 0.26 | 0.02 | 0.42 | 0.06 | 0.41 | 0.51 |
| P79 | F | Eng | 12 | 1.00 | 0.16 | 1.00 | 1.00 | 1.00 | 0.83 | 0.90 | 1.00 | 0.40 | 0.08 | 0.00 | 0.00 | 0.00 | 0.17 | 0.10 | 0.16 |
| P80 | F | Eng | 14 | 1.00 | 0.12 | 0.88 | 0.75 | 1.00 | 0.92 | 0.92 | 0.83 | 0.40 | 0.04 | 0.12 | 0.25 | 0.42 | 0.08 | 0.08 | 0.31 |
| P81 | F | Span | 3 | 1.00 | 0.04 | 0.70 | 0.55 | 0.42 | 0.67 | 0.61 | 0.60 | 1.00 | 0.04 | 0.30 | 0.45 | 0.92 | 0.33 | 0.39 | 0.59 |
| P82 | M | Eng | 3 | 1.00 | 0.04 | 0.76 | 0.50 | 1.00 | 1.00 | 0.84 | 1.00 | 0.60 | 0.04 | 0.24 | 0.50 | 1.00 | 0.00 | 0.16 | 0.67 |
| P83 | F | Eng | 0 | 1.00 | 0.04 | 0.93 | 0.94 | 1.00 | 0.94 | 0.68 | 1.00 | 0.80 | 0.04 | 0.07 | 0.06 | 0.75 | 0.06 | 0.32 | 1.00 |
| P84 | F | Eng | 13 | 1.00 | 0.12 | 0.87 | 0.89 | 1.00 | 1.00 | 0.71 | 1.00 | 0.80 | 0.12 | 0.13 | 0.11 | 0.67 | 0.00 | 0.29 | 0.90 |
| P85 | N/A | Eng | 0 | 1.00 | 0.12 | 0.06 | 0.94 | 0.83 | 1.00 | 0.75 | 1.00 | 1.00 | 0.04 | 0.94 | 0.06 | 0.83 | 0.00 | 0.25 | 0.83 |

Scores are expressed as proportions of ^a^time (for exposure, pre-ABI and post-ABI use, and educational history in L1 and L2), ^b^confidence in L1 and L2, and ^c^family and self-rated proficiency in L1 and L2.

*Proportion values estimated via multivariate imputation by chained equations using the MICE package in R.

BPWA = bilingual people with aphasia; F = female; M = male; L1 = first-acquired language; L2 = second-acquired language; Span = Spanish; Eng = English; AoA = L2 age of acquisition (expressed in years); Pre = pre-acquired brain injury (ABI) metrics; Post = post-acquired brain injury (ABI) metrics; LAR = language ability rating; Family Prof. = family proficiency; Edu. = education; Env. = environment; Expo. = exposure; Conf. = confidence.

**
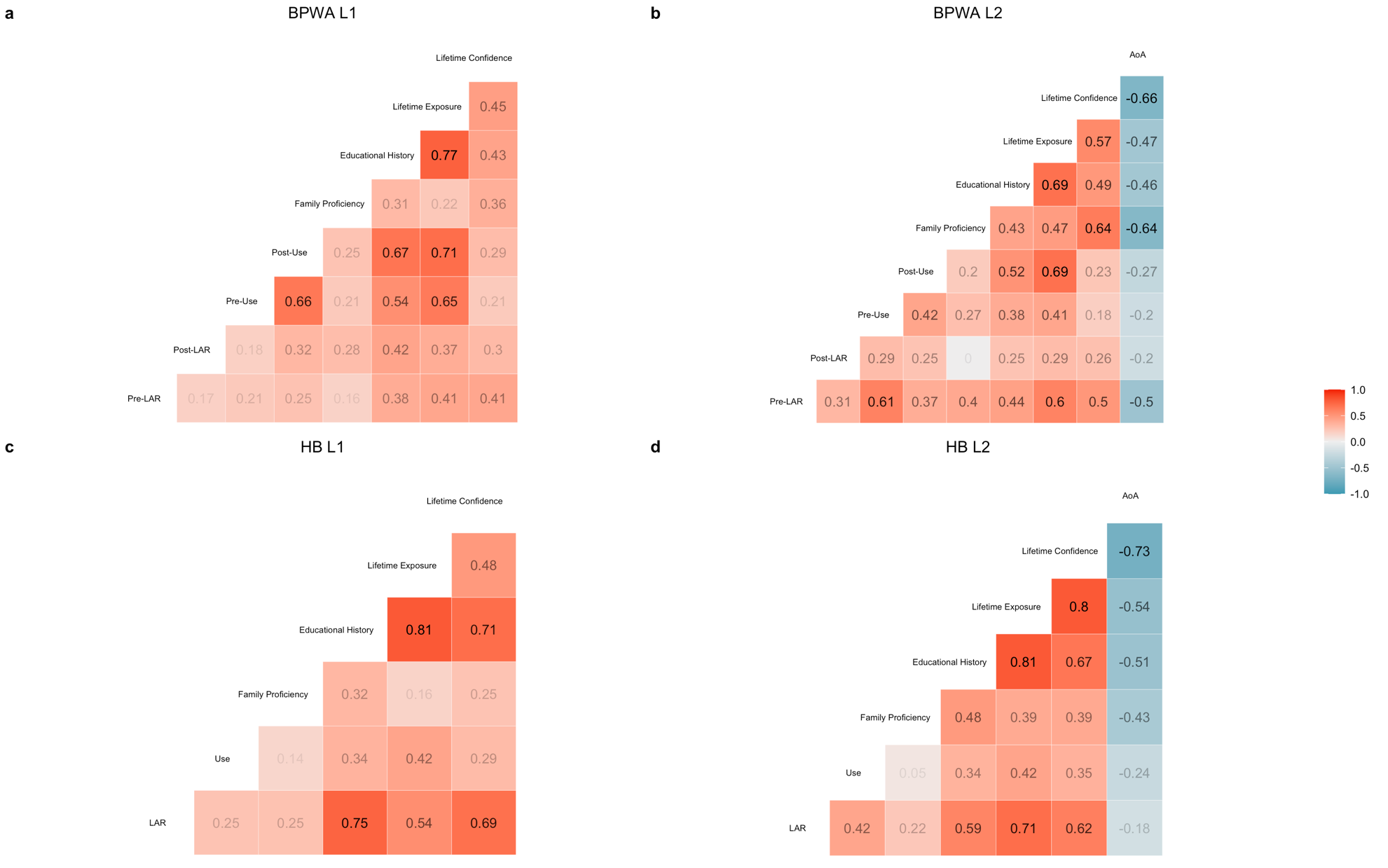
**

**Supplementary Figure 1. Correlation matrices of all included LUQ variables for both HB and BPWA in L1 and L2.**

(a) Correlation matrix for L1 variables in HB. (b) Correlation matrix for L2 variables in HB. (c) Correlation matrix for L1 variables in BPWA. Note pre- and post-ABI distinctions. (d) Correlation matrix for L2 variables in BPWA. Note pre- and post-ABI distinctions. More saturated colors signify greater correlation. Red indicates positive correlation and blue indicates negative correlation. HB = Healthy bilingual; BPWA = Bilingual people with aphasia; L1 = first-acquired language; L2 = second-acquired language; LAR = Language Ability Rating; AoA = L2 Age of acquisition.


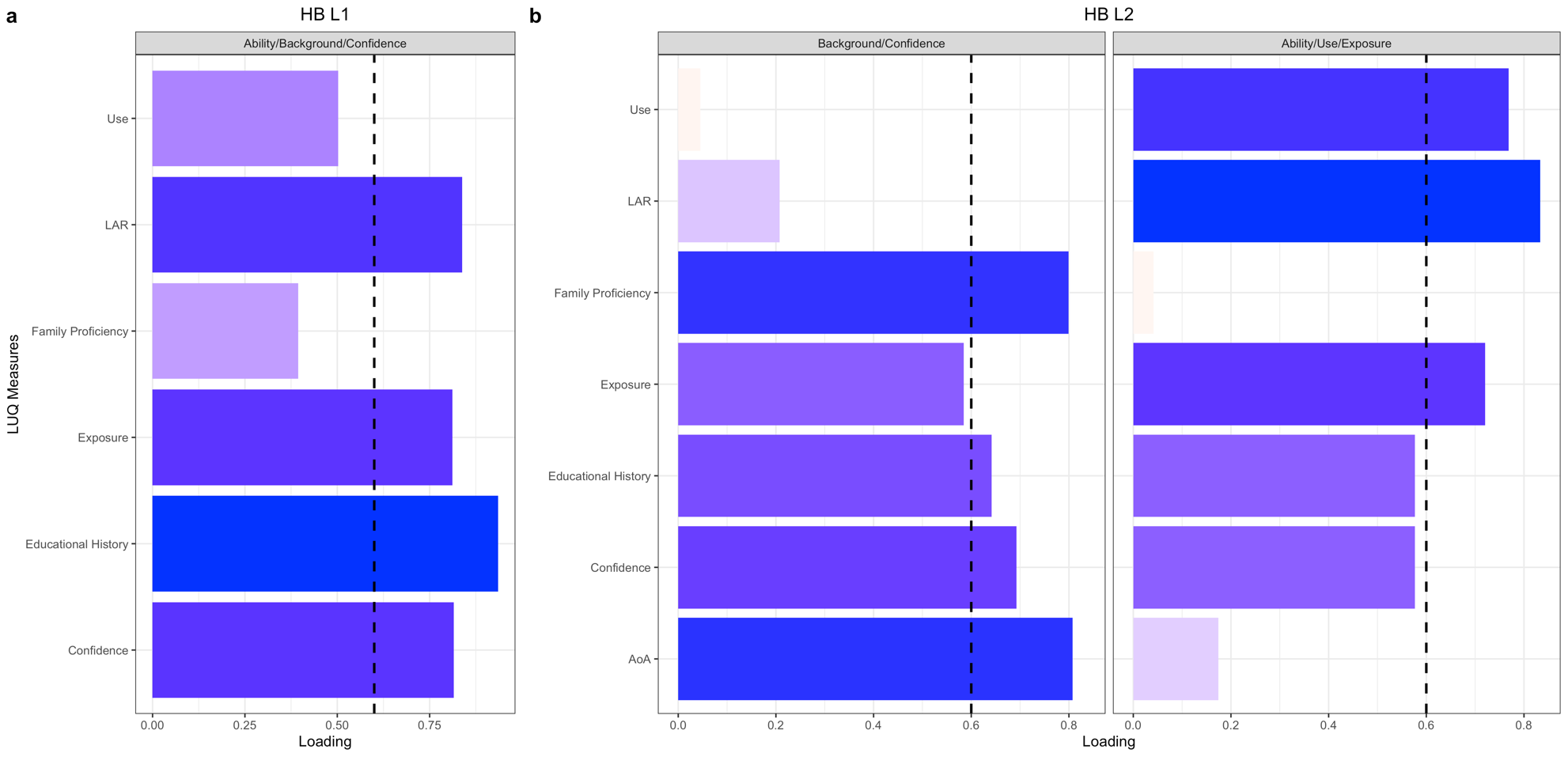


**Supplementary Figure 2. PCA results on HB Language Use Questionnaire metrics in L1 and L2.**

(a) One-component solution for L1 variables and their corresponding loadings in HB. (b) Two-component solution for L2 variables and their corresponding loadings in HB. Each panel shows component loadings of each LUQ measure on the named component included in analysis of HB metrics. Longer and more saturated bars correspond to higher loadings. The hash line represents the recommended threshold (.60) at which measures are considered for interpretation of the component. HB = Healthy bilingual; L1 = first-acquired language; L2 = second-acquired language; LAR = Language Ability Rating; AoA = L2 Age of acquisition.


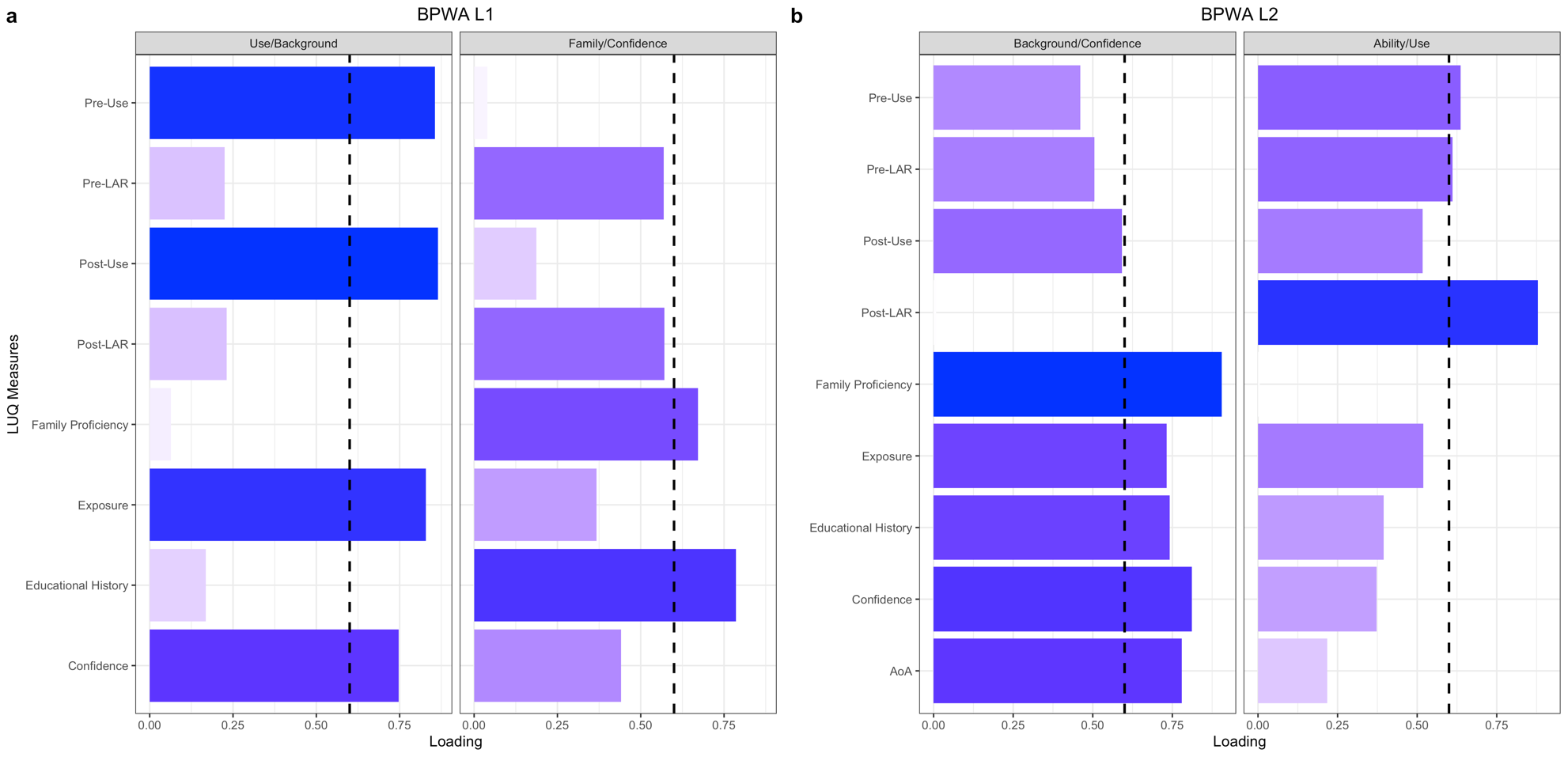


**Supplementary Figure 3. PCA results on BPWA Language Use Questionnaire metrics in L1 and L2.**

(a) Two-component solution for L1 variables and their corresponding loadings in BPWA. (b) Two-component solution for L2 variables and their corresponding loadings in BPWA. Each panel shows component loadings of each LUQ measure on the named component included in analysis of BPWA metrics. Longer and more saturated bars correspond to higher loadings. The hash line represents the recommended threshold (.60) at which measures are considered for interpretation of the component. BPWA = Bilingual people with aphasia; L1 = first-acquired language; L2 = second-acquired language; LAR = Language Ability Rating; AoA = L2 Age of acquisition.
